# Clinical characteristics of *Campylobacter* bacteremia: a multicenter retrospective study

**DOI:** 10.1038/s41598-022-27330-4

**Published:** 2023-01-12

**Authors:** Yuki Otsuka, Hideharu Hagiya, Misa Takahashi, Shinnosuke Fukushima, Ruri Maeda, Naruhiko Sunada, Haruto Yamada, Masayuki Kishida, Koji Fujita, Fumio Otsuka

**Affiliations:** 1 Department of General Medicine, Okayama University Graduate School of Medicine, Dentistry and Pharmaceutical Sciences, 2-5-1 Shikata-Cho, Kita-Ku, Okayama, 700-8558 Japan; 2 Department of General Internal Medicine, Okayama City Hospital, Okayama, Japan; 3 Department of General Medicine and Infectious Disease, Tsuyama Chuo Hospital, Tsuyama, Japan; 4 Department of Clinical Laboratory, Okayama City Hospital, Okayama, Japan

**Keywords:** Clinical microbiology, Gastrointestinal diseases

## Abstract

*Campylobacter* species are the pathogens of the intestinal tract, which infrequently cause bacteremia. To reveal the clinical characteristics of *Campylobacter* bacteremia, we performed a retrospective, multicenter study. Patients diagnosed with *Campylobacter* bacteremia in three general hospitals in western Japan between 2011 and 2021 were included in the study. Clinical, microbiological, and prognostic data of the patients were obtained from medical records. We stratified the cases into the gastroenteritis (GE) and fever predominant (FP) types by focusing on the presence of gastrointestinal symptoms. Thirty-nine patients (24 men and 15 women) were included, with a median age of 57 years and bimodal distribution between those in their 20 s and the elderly. The proportion of GE and FP types were 21 (53.8%) and 18 (46.2%), respectively. Comparing these two groups, there was no significant difference in patient backgrounds in terms of sex, age, and underlying diseases. *Campylobacter jejuni* was exclusively identified in the GE type (19 cases, 90.5%), although other species such as *Campylobacter fetus* and *Campylobacter coli* were isolated in the FP type as well. Patients with the FP type underwent intravenous antibiotic therapy more frequently (47.6% vs. 88.9%), and their treatment (median: 5 days vs. 13 days) and hospitalization (median: 7 days vs. 21 days) periods were significantly longer. None of the patients died during the hospitalization. In summary, we found that nearly half of the patients with *Campylobacter* bacteremia presented with fever as a predominant manifestation without gastroenteritis symptoms.

## Introduction

*Campylobacter* species are microaerophilic, gram-negative, helical-shaped bacteria found in the gastrointestinal tract of many animals^1–3^. *Campylobacter jejuni* accounts for most cases of campylobacteriosis in humans, followed by *Campylobacter coli* and *Campylobacter fetus*^3^. *Campylobacter* infections are typically caused by the ingestion of undercooked chicken, which is a major etiology of infectious gastroenteritis^4^. Bacteremia reportedly occurs in less than 1% of *Campylobacter* infections^5,6^. Nevertheless, it is clinically notable as the disease can develop severe complications, such as endocarditis, infectious aneurysm, osteomyelitis, and pyogenic arthritis^7^.

Meanwhile, because of its rarity, limited clinical reports are available on extraintestinal infections caused by *Campylobacter* species. Although immunocompromised patients, such as those with malnutrition, hematologic and hepatic diseases, and human immunodeficiency virus infection, have been reported to be at risk for *Campylobacter* bacteremia^8^, few studies have revealed their clinical and laboratory characteristics or prognostic factors. In particular, bacteremia without gastrointestinal manifestations is difficult to diagnose^9^, and there is insufficient evidence in such patients. Hence, the purpose of this study was to clarify the clinical characteristics and prognostic factors of *Campylobacter* bacteremia, especially focusing on the presence of gastrointestinal symptoms.

## Materials and methods

### Study design

This was a retrospective study using data from patients with *Campylobacter* bacteremia diagnosed over a decade from April 2011 to March 2021 at the following three general hospitals in western Japan: Okayama University Hospital, Okayama City Hospital, and Tsuyama Chuo Hospital. The background (age, years, month of onset, and underlying diseases), exposure history to a risk food or person, vital signs, symptoms (gastrointestinal manifestations and fever), time from onset to blood culture sampling, pathogenic organisms, laboratory test results on admission (complete blood counts and biochemistry), treatment (intravenous therapy and treatment periods), and prognosis (intensive care unit [ICU] admission rate and hospitalization periods) were extracted from medical records without personally identifiable information. Based on the National Studies of Acute Gastrointestinal Illness’ criteria^10^, patients with at least one episode of diarrhea and/or vomiting were defined as “GE (gastroenteritis) type,” while the remaining patients who had no diarrhea or vomiting in their whole episodes were considered “FP (fever predominant) type.” The shock index of each patient was calculated by dividing pulse rate by systolic blood pressure^11^. Clinical and laboratory parameters of the two types were compared.

### Blood culture and microbiological identification

Each hospital was equipped with in-house microbiology laboratories containing automated blood culture systems: the BD BACTECTM™ FX system (Becton, Dickinson, and Co., NJ, USA) at Okayama University Hospital and Okayama City Hospital, and the BACT/ALERT® VIRTUO® R3.0 System (bioMérieux Japan Ltd., Tokyo, Japan) at Tsuyama Chuo Hospital. At Okayama University Hospital, bacterial strains were identified using API® CAMPY (bioMérieux Japan Ltd., Tokyo, Japan) during 2011–2014 and MALDI Biotyper (Bruker Daltonics GmbH & Co. KG, Bremen, Germany) during 2014–2021. At Tsuyama Chuo Hospital, strains were initially identified based on bacterial metabolic properties, followed by the VITEK MS system (bioMérieux Japan Ltd., Tokyo, Japan) (2011–2017) and MALDI Biotyper (Bruker Daltonics GmbH & Co. KG, Bremen, Germany) (2017–2021) were used. At Okayama City Hospital, the VITEK 2 system (bioMérieux Japan Ltd., Tokyo, Japan) was used during the entire study period.

### Statistical analyses

The data were analyzed using Fisher’s test and Mann–Whitney U test, as appropriate, to determine differences. The log-rank test was used to estimate the difference in hospitalization period between the two types. All tests were two-sided, and *p*-values less than 0.05 were considered statistically significant. All statistical analyses were performed using EZR, version 1.55 (Saitama Medical Center, Jichi Medical University, Saitama, Japan), a graphical user interface for R (The R Foundation for Statistical Computing, Vienna, Austria)^12^.

### Ethics

Information regarding the present study was provided on the website of the Okayama University Hospital, and patients who wished to opt out were offered this opportunity. This study was approved by the Ethics Committees of Okayama University Hospital (No. 1908-056) as well as Okayama City Hospital (No. 3-22) and Tsuyama Chuo Hospital (No. 572), and adhered to the Declaration of Helsinki. Need of informed consent from the patients was waived by the Ethics Committees of Okayama University Hospital because the individual data was fully anonymized. All authors had access to the study data and reviewed and approved the final manuscript.

## Results

### Patient characteristics (Table 1)

**Table 1:** Clinical and microbiological summary of patients with *Campylobacter* bacteremia.

	Total n = 39	GE type n = 21	FP type n = 18	*P* value
**Patient background**				
**Sex**				
Male (%)	24 (61.5)	13 (61.9)	11.1 (61.1)	1.00
**Median age** (IQR)	57 (28–75)	40 (25–77)	58.5 (48.75–72)	0.48
**Underlying diseases** (%)	21 (53.8)	9 (42.9)	12 (66.7)	0.20
Malignant diseases (%)	5 (12.8)	2 (9.5)	3 (16.7)	0.20
Liver diseases (%)	5 (12.8)	1 (4.6)	4 (22.2)	0.16
Kidney diseases (%)	4 (10.3)	2 (9.5)	2 (11.1)	1.00
Blood diseases (%)	8 (20.5)	4 (19.0)	4 (22.2)	1.00
Diabetes mellitus (%)	8 (20.5)	4 (19.0)	4 (22.2)	1.00
Under steroid therapy (%)	5 (12.8)	2 (9.5)	3 (16.7)	0.65
Collagen diseases (%)	2 (5.1)	1 (4.8)	1 (5.6)	1.00
Under chemotherapy (%)	6 (15.4)	4 (19.0)	2 (11.1)	0.67
**Exposure history to possibly contaminated food (%)**	8 (20.5)	7 (33.3)	1 (5.6)	0.049
**Symptoms and vital sign**				
Diarrhea (%)	21 (53.8)	21 (100)	0	< 0.01
Vomiting (%)	5 (12.8)	5 (23.8)	0	0.050
Stomachache (%)	17 (43.6)	15 (71.4)	2 (11.1)	< 0.01
Fever (%)	35 (89.7)	20 (95.2)	15 (83.3)	0.32
Shock index (IQR)	0.83 (0.63–0.99)	0.84 (0.68–1.08)	0.71 (0.62–0.87)	0.22
**Diagnosis**				
Time from the onset to the blood culture sampling (days) (IQR)	1 (0–2)	1 (1–2)	0 (0–2)	0.86
**Species (%)**				
*Campylobacter jejuni*	27 (69.2)	19 (90.5)	8 (44.4)	< 0.01
*Campylobacter fetus*	5 (12.8)	0	5 (27.8)	
*Campylobacter coli*	4 (10.3)	1 (4.8)	3 (16.7)	
*Campylobacter lari*	1 (2.6)	0	1 (5.6)	
*Campylobacter ureolyticus*	1 (2.6)	1 (4.8)	0	
Unidentified	1 (2.6)	0	1 (5.6)	
**Treatment**				
Intravenous therapy (%)	26 (66.7)	10 (47.6)	16 (88.9)	< 0.01
Treatment periods, days (IQR)	9 (4–15)	5 (3–10)	13 (6–18)	0.02
**Prognosis**				
Hospitalization (%)	35 (89.7)	18 (85.7)	17 (94.4)	0.61
Hospitalization periods, days (IQR)	12 (6–30)	7 (3–17)	21 (11–33)	< 0.01
ICU admission (%)	2 (5.1)	1 (4.8)	1 (5.6)	1.00
Death	0	0	0	-

The data were analyzed by the Mann-Whitney U test and Fisher’s test, as appropriate.

*FP* fever predominant, *GE* gastroenteritis, *IQR* interquartile range, *ICU* intensive care unit.

**Figure 1:**
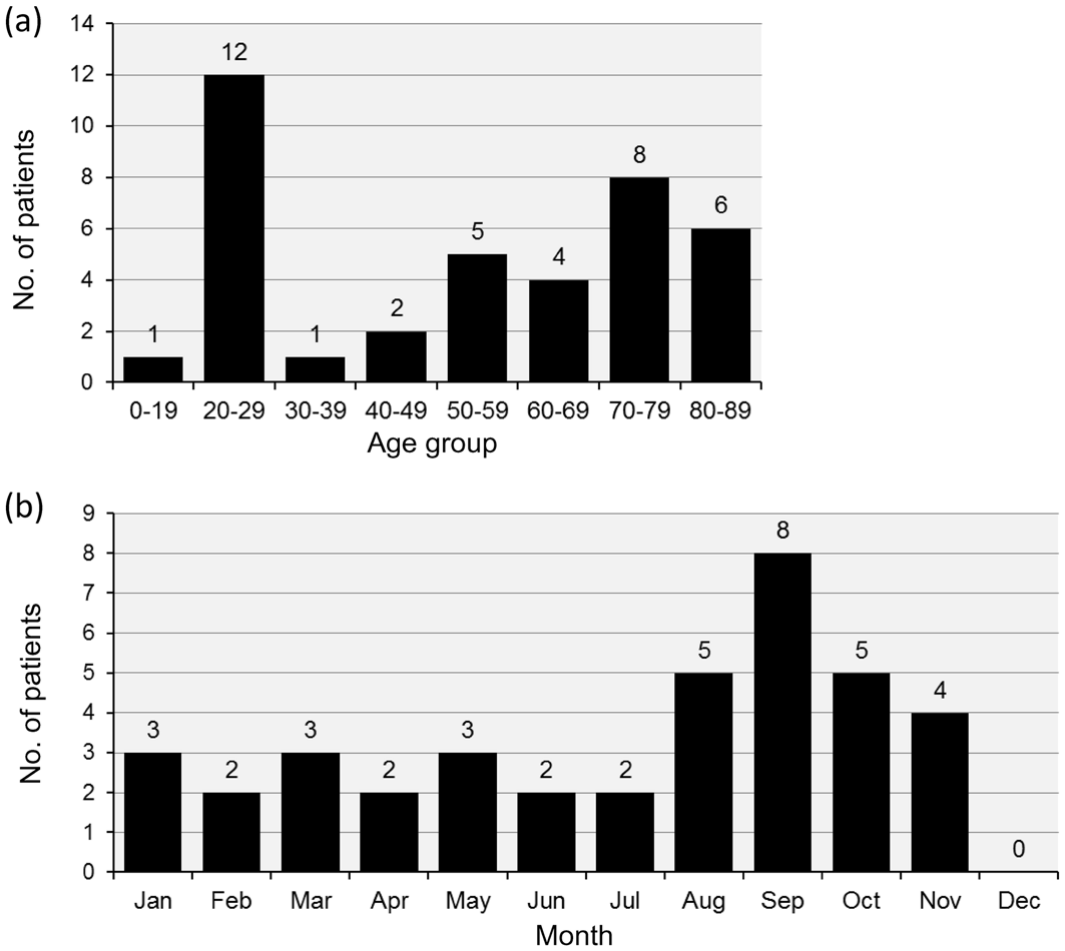
Incidence of *Campylobacter* bacteremia by age group and month. Patient age groups were distributed bimodally between the 20s and the elderly (**a**). An incidence peak was observed in September (**b**).

Among the 39 patients with *Campylobacter* bacteremia, 24 were men and 15 were women. The median age was 57 years (interquartile range [IQR]:28.0–74.5), and the distribution was bimodal with cases peaking sharply in those aged in their 20s and broadly in older adults aged over 50 years (Fig. 1a). The incidence peaked in September (Fig. 1b). Only eight patients (20.5%) reported an obvious exposure history, such as eating raw foods or having contact with an unhealthy individual. Diarrhea, vomiting, stomach ache, and fever were observed in 21 (53.8%), 5 (12.8%), 17 (43.6%), and 35 (89.7%) patients, respectively. *C. jejuni, C. fetus, C. coli, C. lari, and C. ureolyticus* were identified in 27 (69.2%), 5 (12.8%), 4 (10.3%), 1 (2.6%), and 1 (2.6%) patients, respectively. Specific species were unidentifiable in one case (Table 1).

### Treatment and prognosis

Thirty-five patients (89.7%) were hospitalized. Three patients (8.6%) were readmitted to other hospitals for rehabilitation and the remaining 32 (91.4%) were discharged to their homes. In total, only two patients (5.1%) were admitted to ICUs for postoperative management of an infected aneurysm and septic shock. Overall, 37 patients (94.9%) were treated with antibiotics and 26 (66.7%) received intravenous therapy. As a complication, infected aneurysm of the iliac artery and subcutaneous soft tissue infection were observed in one and five cases, respectively.

### GE type vs. FP type

Comparisons of the clinical and microbiological characteristics of the patients are provided in Table 1. Among the 39 cases, the number of patients in GE type and FP type was 21 (53.8%) and 18 (46.2%), respectively. Comparing these two types, there was no significant difference in the patients’ backgrounds, such as sex, age, and underlying diseases, whereas an exposure history was identified more frequently in the GE type. The shock index was also not significantly different between the two types. In addition to diarrhea and vomiting, stomachache was significantly predominant in the GE type: 15 patients (71.4%) with GE type vs. 2 patients (11.1%) with FP type. Similarly, fever was observed at the time of hospital visit in 20 patients (95.2%) with the GE type and in 15 patients (83.3%) with the FP type. Among 18 FP type patients, three suffered from lower limb cellulitis, two suffered from femoral osteomyelitis, and two suffered from febrile neutropenia associated with lymphoma. Times from the onset to blood culture sampling did not differ between the two types: 1 (IQR: 1–2) days in the GE type and 0 (IQR: 0–2) days in the FP type (*P* = 0.86). Microbiologically, *C. jejuni* was exclusively identified in the blood sample of up to 19 patients (90.5%) in the GE type, although other species such as *C. fetus* and *C. coli* caused bacteremia in the FP type as well. Comparing the laboratory results, hypokalemia and elevated serum creatinine levels were significantly associated with GE type (Table 2).

**Table 2:** Comparison of laboratory data on admission between GE type and FP type Campylobacter bacteremia.

	GE type n = 21	FP type n = 18	*P* value
WBC (/uL)	10,990 (4680–11,800)	11,470 (8305–13,918)	0.54
Hgb (g/dL)	13.7 (11.4–15.0)	12.4 (9.78–13.2)	0.65
Plt (10^3/uL)	18.1 (14.1–21.3)	17.2 (7.0–27.2)	0.81
CRP (mg/dL)	5.60 (3.83–10.5)	2.65 (1.38–8.76)	0.13
Na (mEq/L)	138.0 (136.0–138.0)	137.5 (135.3–140.0)	0.93
K (mEq/L)	3.6 (3.5–3.7)	3.8 (3.7–3.9)	< 0.01
Cl (mEq/L)	101 (99–106)	101 (99–104)	0.80
UN (mg/dL)	14.9 (9.9–17.9)	15.9 (11.0–19.9)	0.50
Cr (mg/dL)	0.95 (0.74–1.23)	0.68 (0.59–0.86)	0.014
UN/Cr ratio	15.0 (11.6–19.5)	18.6 (15.6–27.1)	0.011
AST (IU/L)	22 (16–29)	27 (19–38)	0.28
ALT (IU/L)	17 (11–31)	22 (12–53)	0.60
LDH (IU/L)	205 (178–293)	208 (184–293)	0.77

The data were analyzed by the Mann–Whitney U test. Median and interquartile range are demonstrated.

*ALT* alanine amino transferase, *AST* aspartate aminotransferase, *Cl* chloride, *Cr* creatinine, *CRP* C-reactive protein, *Hgb* hemoglobin, *K* potassium, *LDH* lactate dehydrogenase, *Plt* platelets, *Na* sodium, *UN* urea nitrogen, *WBC* white blood cells, *GE* gastroenteritis, *FP* fever predominant.

**Figure 2:**
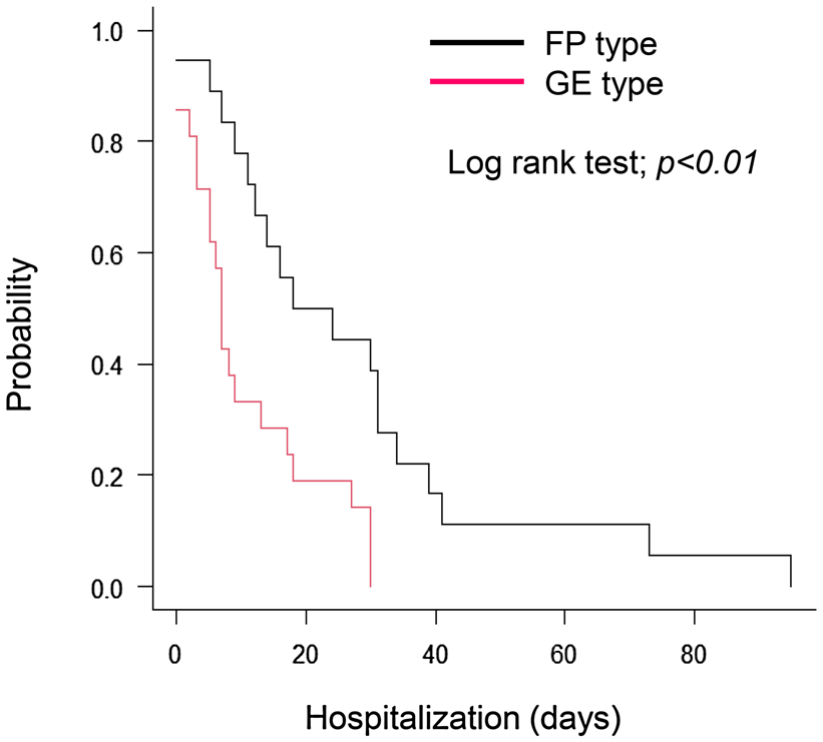
Kaplan–Meier curve for hospitalization periods. Comparison of gastroenteritis (GE) type and fever predominant (FP) type. *Campylobacter* bacteremia showed that hospitalization days were significantly longer in the FP type (*P* < 0.01).

Patients with the FP type underwent intravenous antibiotic therapy more frequently than those with GE type: 10 (47.6%) vs. 16 (88.9%) (*P* < 0.01). The treatment period was significantly longer in the FP group (13 days [IQR: 6–18]) than in the GE group (5 days [IQR: 3–10]) (Table 1). ICU admissions were observed in one case each for the GE and FP types. The median duration of hospitalization was significantly longer in the FP group (21 days [11–33]) than in the GE group (7 days [IQR: 3–17]). Log-rank test analysis also resulted in longer hospitalization in the FP-type (*P* < 0.01) (Fig. 2). None of the patients died during the hospitalization.

## Discussion

In this study, we investigated the clinical and microbiological characteristics of *Campylobacter* bacteremia in multiple medical centers across Japan. Clinically, our data highlighted that *Campylobacter* bacteremia inflicts not only the elderly, but also young patients in their twenties. Unlike *Salmonella*, which is known not only as a causative bacterium of gastroenteritis but also a cause of fever of unknown origin (FUO)^13^, *Campylobacter* infections have not been recognized to present fever as a predominant symptom. Notably, our findings suggest that patients with *Campylobacter* bacteremia may present with fever as a predominant manifestation without gastroenteritis symptoms. Despite similar clinical backgrounds, patients with FP type *Campylobacter* bacteremia required intensive treatment with antibiotics and were hospitalized for long periods. Microbiologically, nearly 90% of GE type *Campylobacter* bacteremia was caused by *C. jejuni*, while FP type *Campylobacter* bacteremia was caused by various species of *Campylobacter*.

Our efforts revealed intriguing new clinical features of *Campylobacter* bacteremia. First, although *Campylobacter* bacteremia is commonly reported to develop in the elderly^14^, in the present study, the highest number of cases was observed in those aged 20–29 years. Consistent with our own results, infected young patients without any particular risk factors developing *Campylobacter* bacteremia has been reported in recent studies^5,6^. Second, the underlying medical backgrounds of patients with GE- and FP type *Campylobacter* bacteremia were not significantly different. Although typical campylobacteriosis is accompanied by gastroenteritis symptoms, vulnerable patients with immunocompromising factors are reported to have fewer enteritis symptoms^15^. Another retrospective cohort study also suggested that primary bacteremia without gastroenteritis occurs, especially in immunocompromised patients^8^. However, our patients with GE- and FP type diseases showed similar clinical backgrounds, indicating that patients without particular underlying medical conditions can also present with FUO-like *Campylobacter* bacteremia. Thus, blood culture sampling remains essential even for previously healthy individuals, to diagnose *Campylobacter* bacteremia. Considering that blood cultures are not routinely obtained for patients with gastroenteritis in general practice, there may be many more bacteremia patients than are currently recognized^16,17^.

Laboratory data analysis would not be informative for distinguishing between patients with GE type and FP type *Campylobacter* bacteremia. Compared with patients with the FP type, those with the GE type showed elevated serum creatinine levels (median; 0.95 mg/dL vs. 0.68 mg/dL). This was reasonable considering that such patients were more dehydrated because of gastroenteritis-associated vomiting and diarrhea. Otherwise, there were no remarkable points to be addressed in routine laboratory data.

The unique features of *Campylobacter* species should also be considered. *C. jejuni* is the most common species in human infections and frequently causes gastroenteritis, but fewer complications develop outside the gastrointestinal tract^14,16^. Notably, our data revealed that *C. jejuni* potentially causes bacteremia and demonstrates a FUO-like presentation as well, which has also been pointed out in the preceding literature^5,6^. Despite being a rare species for human infection, *C. fetus* is more common in agriculture or livestock with cattle and sheep as the main reservoirs and tends to cause bacteremia without apparent gastroenteritis in humans^18,19^. Thus, the isolation of various *Campylobacter* species other than *C. jejuni* in FP type was consistent with previous findings. This finding can be partly attributed to the improvement in bacterial identification in microbiological laboratories^20^.

Previous studies estimated the mortality rate of *Campylobacter* bacteremia to be 4–28%^5,21^, whereas no hospital deaths were observed in our study. Although details of the direct causes of death in these reports were unavailable and it is difficult to simply compare our cohort with those included in preceding studies, our study also included some patients with complicated, immunocompromised underlying diseases. Delayed appropriate antimicrobial therapy, as a consequence of a lack of clinical symptoms, is reportedly associated with high mortality, defining the prognosis of *Campylobacter* bacteremia^14,22^. However, the time from onset to diagnosis did not differ between GE and FP types in the present study. Further studies with larger case numbers are needed to clarify this point.

One strength of this study is that we stratified the cases from a clinical perspective based on the presence of symptoms of gastroenteritis. Previous studies on *Campylobacter* infections have focused on the differences in bacterial species, and our results should thereby provide valuable additional information for clinicians. Nevertheless, this study has some limitations. First, due to the retrospective nature of the study, there were some missing values in clinical and microbiological data. Due to the study design, GE symptoms may have been overlooked in some cases of FP type, which cannot be optimized any further. Second, a selection bias may have existed because only blood culture-positive cases were included. Third, this study could not provide epidemiological data, such as the incidence or prevalence of bacteremia cases among *Campylobacter* infections. Forth, there may be interviewer bias in examining exposure history in the GE type. Finally, due to the limited number of cases, we could not apply multivariate analysis to identify clinical and microbiological factors to distinguish between ancillary information of the GE and FP types. Despite these limitations, we believe that our study provides additional data for understanding *Campylobacter* bacteremia.

In conclusion, we revealed the clinical and microbiological characteristics of *Campylobacter* bacteremia through a multicenter investigation. Nearly half of the patients showed fever-predominant manifestations, and their clinical backgrounds were comparable to those of patients with gastroenteritis symptoms. However, patients with fever-predominant *Campylobacter* bacteremia require longer treatment and hospitalization periods. Clinicians should recognize that patients with *Campylobacter* infections can present with fever alone as those with FUO do, even in the absence of conventional GE symptoms.

## Data Availability

Data in detail will be available if requested to the corresponding author.
